# Containment and therapeutic relationships in acute psychiatric care spaces: the symbolic dimensions of doors

**DOI:** 10.1186/s12888-021-03607-2

**Published:** 2022-01-04

**Authors:** Evdokia Missouridou, Evangelos C. Fradelos, Emmanouel Kritsiotakis, Polyxeni Mangoulia, Eirini Segredou, Ioanna V. Papathanasiou

**Affiliations:** 1 Department of Nursing, Faculty of Health and Caring Professions, University of West Attica, Saint Spiridonos 12243, Egaleo, Athens, Greece; 2 Community Nursing Lab, Department of Nursing, University of Thessaly, Larissa, Greece; 3 Psychiatric Department, General State Hospital “Sismanoglio”, Marousi, Greece; 4 Psychiatric Liaison Unit, General State Hospital “Evangelismos”, Athens, Greece; 5 Alcohol Treatment Unit, Psychiatric Hospital of Attica, Chaidari, Greece

**Keywords:** Acute psychiatric care, Open doors, Door locking practice, Nurses, Greece

## Abstract

**Background:**

There is an increasing trend of door locking practices in acute psychiatric care. The aim of the present study was to illuminate the symbolic dimensions of doors in Greek mental health nurses’ experiences of open and locked working spaces.

**Results:**

A sequential mixed-method designexplored the experiences of nurses working in both open and locked psychiatric acute care units. Participants experiences revealed four types of doors related to the quality of recovery-oriented care: (a) the open door, (b) the invisible door, (c) the restraining door, and (d) the revolving door. Open doors and permeable spacesgenerated trust and facilitated the diffusion of tension and the necessary perception of feeling safe in order to be involved in therapeutic engagement. When the locked unit was experienced as a caring environment, the locked doors appeared to be “invisible”. The restraining doors symbolized loss of control, social distance and stigma echoing the consequences of restrictingpeople’s crucial control over spaceduring the COVID-19 pandemicin relation toviolence within families, groups and communities. The revolving door (service users’ abscondence/re-admission) symbolised the rejection of the offered therapeutic environment and was a source of indignation and compassion fatigue in both open and locked spaces attributed to internal structural acute care characteristics (limited staffing levels, support, resources and activities for service users) as well as ‘locked doors’ in the community (limited or no care continuity and stigma).

**Conclusions:**

The impact of COVID-19 restrictions on people’s crucial control of space provides an impetus for erecting barriers masked by the veil of habit and reconsidering the impact of the simple act of leaving the door open/locked to allow both psychiatric acute care unit staff and service users to reach their potential.

## Introduction

Persons with mental health problems often experience distressing feelings culminating in a crisis which neither themselves nor their families can contain prior to seeking refuge to a bigger system of relationships in acute psychiatric care spaces. These spaces constitute a temporary escape, ‘a third place’ [1] of emotional containment and refuge in varied distance from family (the first system) or community (the second system) and a space apart from the pressures of the latter systems. Physical and emotional containment can be attained through containment measures as well as relationships which are founded in trust and have the capacity to bear feelings too difficult for the people to manage on their own [2]. Mental health service users stress that relationships of trust and respect, in which they are listened to, are of great importance to them [3]. Establishing a therapeutic alliance depends on several factors such as the clinician’s ability to have empathy and the patient’s degree of underlying psychopathology and might be possible within minutes or could take years [4]. Peplau [5] described therapeutic relationships as the foundation of psychiatric nursing because they create feelings of being held together and of being safe. She also urged nurses to focus on persons rather than patients and ‘give up the notion of a disease, such as schizophrenia and to think exclusively of patients as persons’ [6]. Understanding the person and their experiences, facilitating growth, therapeutic use of self, choosing the right approach and authoritative vs. emotional containment, emerged as the “Principles of Engagement” in acute mental health wards [7]. Positive ward atmosphere increases service users’ satisfaction and strengthens the therapeutic alliance [8, 9]. Moreover, a good therapeutic relationship is associated with better health outcomes for patients, enhances the effectiveness of interventions in inpatient mental health care, and improves both patients’ well-being and experience [10]. Cultural norms, subjective ways in which people interpret and use spaces, expectations and narratives can play an important role in shaping people’s experiences of space [11, 12] while physical and built environments, social conditions and human perceptions contribute in combination in promoting healing at given ‘therapeutic landscapes’ [13].

The study of Roviralta-Vilella et al. [14] shows that the factors associated with higher-quality therapeutic relationship in mental health units are a more favourable nurse practice environment and, specifically, the presence of more foundations for quality nursing care, together with higher academic qualifications and longer nurse experience. The lack of time is seen as the major obstacle to achieving the therapeutic relationship, both by nurses and by patients. Another limiting factor in the therapeutic relationship is an insecure setting [15]. In some countries spaces have been specially set aside for contact with patients (‘patient-protected time’), nevertheless, there continue to be problems, because of personnel shortages or a lack of support of supervisors [16]. Of course this alone will not eliminate the unpredictability of the surrounding, given that are acute psychiatric units, so nurses have to work hard to ensure that it does not entail insecurity in the hospital unit [15]. The review of Moreno-Poyato et al. [16] points to the need for nurses to have greater organizational support, the importance of promoting effective teamwork and the existence of a nursing model within the units. However, both the organizational climate, culture, safety and physical infrastructure of a ward, alongside nurses’ and patients’ own personal resources may either positively or negatively influence engagement. Additionally, an individual’s actual or perceived capabilities, opportunities and motivation drive their ability to overcome the influential factors [7].”

On the other hand, doors constitute boundaries of acute psychiatric care environment which limit or enhance control over space [17]. Door locking constitutes a measure of containing the psychiatric crisis which regulates the ability of both voluntary and involuntary patients to leave psychiatric units [18]. Research on staff, patient and visitors’ perceptions and experiences of door locking practices has identified both advantages and disadvantages in relation to locked environments [19–21]. On the other hand, research on open wards is very limited and focuses mostly on the success of treatment in terms of absconding, aggression and coercive measures rates [22–24] or the implementation of open-door policies in wards with a tradition of locked door policy [25]. Efkemann et al. [8] attempted to illuminate the perceptions and experiences of patients and staff in a mixed method study of wards traditionally operating in an open or locked policy and concluded that ward atmosphere was associated with the door policy status. Similarly, McKeown et al. [26, 27], in an ethnography of coercion in acute psychiatric care in the UK, observed the profound impact of open doors and less rigid demarcations of ward space on therapeutic and social contacts and identified legitimation as a crucial process in professionals’ justifications for door locking practices. In Greece, a recent study of nursing students’ attitudes towards open door policy and restrictive measures concluded that the culture of psychiatry in a locked or open unit with custom restrictive practices socialized students’ views towards the locally dominant pattern of relative evaluations [28]. In contrast, a recent study of Greek nurses’ experiences in open wards [29] and a study of nursing care providers’ experiences in locked wards [30] found that negative and positive feelings about door locking did not appear to match the specific system of practice since participants described how open or locked door practices influenced their professional role. In pursuit of a comparable proximity to the results of these two studies, the authors further examine the symbolic dimensions of doors in mental health nurses’ descriptions of their working environments. Thus, in the study reported here we provide new insights in the descriptive nature of open and locked door practices and the symbolic meanings assigned to doors in acute psychiatric care spaces.

### The Greek context

In Greece, the debate on the concept of recovery [31] is growing steadily [32] in alignment with many other countries in Europe and the US [33] while emphasis is placed on social justice issues [34], the education of mental health professionals [28], service users experiences [35–37] and trauma-informed care and education [34, 38, 39]. Positive initiatives include an anti-stigma movement, the development of societies comprising the families of service users, and a Greek Hearing Voices Network [40]. Nonetheless, the percentage of involuntary admissions in both the two public psychiatric hospitals of Athens (i.e. approximately 54%) and the psychiatric inpatient units of general hospitals (i.e. approximately 35%) is alarmingly high [41] in the context of a long-lasting financial crisis [42].

## Methods

### Aim and study design

The aim of the present study was to describe the symbolic dimensions of doors in mental health nurses’ experiences of their working environments (25-bed acute care units). Person-centered care (medication, psychoeducation and social care) in these environments was provided by a multidisciplinary team for approximately six weeks to three months. A sequential mixed method qualitative approach was employed to allow for triangulation of methods and a final phase of integration of data [43] as well ensuring methodological integrity evaluated by (a) fidelity to the subject matter and (b) utility in achieving research goals [44] as follows:

- Study one: An inductive content analysis qualitative study [29] conducted between May 2017 – November 2017 in six open acute psychiatric care wards. Analysis started immediately after conducting an interview while emerging codes were discussed with the primary researcher prior to conducting another interview. Additionally, saturation issues were discussed with the primary researcher at later stages.
- Study two: A thematic analysis qualitative study [30] in six locked acute psychiatric care wards which began at the completion of study one data collection and was completed on October 2018. Analysis started during the later stages of data collection.

Interviewers were student nurses with a six-month clinical placement in mental health acute care units and were prepared for their research by the first three researchers who were also responsible for their clinical training.

### Participants & procedure

Purposive sampling aiming to achieve an equal participation of male and female participants was used to approach nurses who provide services to service users in open and locked wards. The nurse director of each hospital provided the names of nursing professionals who were available for an interview. The number of professionals interviewed in a unit was limited to two to ensure equal participation of participants from different units. Eleven out of twenty-two participants were male (50%). Participants’ age ranged from 33 to54 years (mean 43.3 years) while their clinical experience ranged from 4 to 28 years (mean 18.1 years). All participants (100%) had a degree in Nursing. One participant held a MSc degree and four participants had acquired a post-graduate specialization in mental health nursing. Individual interviews (thirty to sixty minutes duration) were conducted by three female student nurses supervised by the first author in the context of two research projects completed as part of an undergraduate degree course. Interviewers had a varied support from the second and the third author.

An introductory question (What are the advantages and disadvantages of the open/locked door practices for your nursing care?) generated lively discussions about nurses’ experiences of working in open/locked wards. This was followed by further questions: What are the advantages and disadvantages of the open/locked door practices for the people with mental health problems and your relationship to them? What were your first impressions of working in an open/locked ward when you started working? Have your feelings or the way you think changed since then? A closing question invited participants to offer description of the impact of their working experience on them (How do you think working in an open/locked ward affected you over time?) as well as recommendations that may support their work in future. Questions were open-ended, with probes facilitating rich accounts.

### Ethical approval and conduct

Participants were recruited in the study on a voluntary basis and ground rules around disclosures, respect for participants’ privacy and anonymity were discussed with the participants prior to participation. All participants were informed of their rights to refuse or to discontinue their participation, according to the ethical standards of the Helsinki Declaration of 1983 and signed an informed consent form. The study was approved by the Scientific Committees of the Hospitals included in the study.

### Analysis

In our attempt to understand the richness of the data and to interpret the ‘social reality’ of participants, the process of analysis included open coding, creating categories and abstraction [45]. To ensure the credibility of findings, the first three researchers read independently the transcripts and consensus was reached on the identified themes and subthemes. Confirmability of results was enhanced by data (space and person) triangulation [46] and researcher triangulation. Finally, all participants’ quotations aiming at illuminating authentic closeness to the subject matter have been reported solely in the present article.

In the present study the researchers reflected on personal experiences and pre-understanding that may influence the research process. The first four authors, two female and two male, are mental health nurses sharing a varied commitment to trauma informed care and reduction of coercive care. The first and the fifth author have a systemic and group analytic background respectively. The second and the third authors are currently completing their studies on social and political sciences and worked at the time of the study at locked and open acute care wards respectively at the hospitals involved in the study. The last author is a professor of Community Psychiatric Nursing.

## Results

Nurses’ experiences varied greatly among wards and hospitals and participants described several locked doors (e.g. unit entrance, nurses’ room) as well as open doors (e.g. unit entrance, patient room) in the unit space. The type of ‘door’ appeared to be a central organising element of participants’ experiences in locked and open acute care units. Overall, four types of doors were identified in interview transcripts (a. the open door, b. the invisible door, c. the restraining door, and d. the revolving door), while eighteen sub-themes described their working experiences and perceptions in relation to the four door types in acute psychiatric care units (Table 1).

**Table 1:** Themes and sub-themes

THEMES	SUB-THEMES
The open door	• feeling of freedom and therapeutic atmosphere • trust and collaboration • enhanced socialization • reduced likelihood for aggression and conflict • service users’ empowerment and nurses’ increased self-awareness
The invisible door	• limit setting • safety and privacy • meaningful staff-patient interactions
The restrictive door	• a strong impression of “prison like” environment • difficulty in building trust and therapeutic alliances • conflict and aggressionincidents • stigma • service users’ disempowerment and nurses’compassion fatigue
The revolving door	• service users’ relapse and nurses’compassion fatigue • lack of care continuity • substance misuse • limited resources and containment in the context of multidisciplinary team • guilt and fear of litigation

### Theme 1: the open door

This theme comprised five sub-themes: (a) feeling of freedom and therapeutic atmosphere, (b) trust and collaboration, (c) enhanced socialization, (d) reduced likelihood for aggression and conflict, and (e) service users’ empowerment and nurses’ increased self-awareness.

Participants described the therapeutic atmosphere created by an open-door emphasizing that the feeling of freedom is therapeutic since having opportunities for choices instils hope in an individual about their future. According to interviewees, people can leave at any time and in essence feel independent and free to decide about their care in collaboration with the nurse.

"The open ward offers more freedom to the patients. They are calmer, it is better for them when they have the opportunity to go out. Patients in open wards have options. This also benefits the nurses because we have the cooperation and participation of the patients. They understand that they do not stay in the clinic by force, they are not forced, they stay because they want to get well." (O9)

"You have to convince the patients and not impose yourself on them when patients have the opportunity to leave. This requires great mental strength and abilities. You learn to listen. All they want is someone to listen to their pain and traumas … " (O2)

Trust in therapeutic relationships is greatly dependent on the trust being given to people indirectly by an open-door. According to participants, being trusted enhances service users’ self-determination and self-confidence leading to their empowerment. An open-door enhances their morale since the open door means for them that they are trusted and that they are able to preserve their dignity as much as possible. Recovery involves collaboration, listening to, learning from and acting upon communications and clarifications on what is important to people. As the latter discuss with nurses their needs and realize the options they have,they feel the nurse close to them, a supporter helping in their recovery rather than an obstacle. Furthermore, they gradually feel confident that the nurse will listen to them, be interested and help.

“The main thing for me is that in the open ward you observe patients and see if they want to stay in treatment. They are becoming aware of their illness, you discuss all this with them, they tell you: ‘I do not feel well’, ‘I want to see the doctor’, ‘I want to change my medication’. The main thing is that they stay because they want to. They have the option or the right to put it better, to leave at any time”. (O3)

“Just because of the freedom of movement, I believe that a two-way relationship of trust is created between the patient and the nurse. The patient thinks that the nurse shows me confidence to go out for a walk, I will trust him/her too. It all works therapeutically.” (O7)

“In the open wards you offer something more human … Patients are tied to you. They want you. They leave and come to greet you…” (O5)

Participants described that contact with the ‘outside world’ and socialization with people from other units works therapeutically for service users and contributes to their good mental health and faster discharge from the hospital. The latter was likened to a ‘small village’.

“In the open wards, the environment is more beautiful, the patients’ energy is channeled. They will go for a walk, they will talk to other patients, they will go out to have a cup of coffee”. (O3)

“In the open wards, the emotional tension is diffused. The patients’ energy is channeled, they get socialized. Even when their conversations do not make sense, for them it is a form of ‘psychotherapy’.” (O4)

Several participants reported that through mental health nursing in open units they gradually gained self-awareness within a particular socio-political context. Typically, they believe that they have become better professionals and better parents in their families. Through daily work they understood their needs and their limits. Limits are also necessary in the treatment of people in the landscape of mental health problems and trauma and require communication as well as to develop the ability to set limits without becoming distant and authoritarian.

“I gradually got to know my limits. You communicate, you care about the patient, you come to understand his/her world, you listen to him/her. You change as a human being.” (O2)

“Through your contact with the patient you discover yourself, and your limits.” (O8)

### Theme 2: the invisible door

The therapeutic benefits of locked doors appeared to be the central organising element of participants’ experiences in some locked units. When the locked unit was experienced as a caring environment, the locked doors appeared to be ‘invisible’. This theme comprised three sub-themes: (a) limit setting, (b) safety and privacy, and (c) meaningful staff-patient interactions.

Several participants reported that limit setting is one of the most important parts of patients’ treatment as well as being fundamental in a successful collaboration between patients and nurses. The practice of locking the door helps a lot in cultivating limit setting and promoting responsible, sensible and prudent decision making. Some nurses noted that physical boundaries enable the patient to internalize the importance of limit setting and self-control in his/her recovery.

“I see people with mental health problems as my children and treat them with the same compassion or strictness. I want to give them the best I can, to understand them, to help them and to teach them not to exceed certain limits.” (L1)

“I believe there is no locked door. The lock is just a virtual constraint and the railings are natural limits which in essence prevent patients from delinquent behavior ... The natural limits used in the locked wards constitute the means of teaching patients to internalize ethical limits and help them to reintegrate in society after being discharged from a psychiatric hospital.” (L8).

According to participants, patients rarely admit that they feel safe in the locked ward and that they do not suffocate. However, several patients have shared with participants that they feel protected and safe behind bars and locked doors for different reasons (the source of threat may be another patient, an unwanted relative, or a symptom of their illness). The gradual attainment of trust within therapeutic relationships contributed to perceiving the environment as primarily caring instead of ‘locked’.

“No matter how much the patients react against the practice of the locked door, both directly and indirectly, after they get better, they thank us and sometimes they apologize for hurting us, for treating us badly or because we just saw them in their worst phase.” (L9).

### Theme 3: the restraining door

This theme comprised five sub-themes: (a) a strong impression of “prison like” environment, (b) difficulty in building trust and therapeutic alliances, (c) conflict and aggression incidents, (d) stigma, and (e) service users’ disempowerment and nurses’ compassion fatigue.

According to some participants, the locked door restrains service users’ freedom to the extent that the latter often liken the locked ward to a ‘prison’. People admitted involuntarily are commonly highly negative with the locked ward describing feelings of imprisonment which in combination with their vulnerable psychiatric condition creates tensions and often makes them more aggressive. Tension in the atmosphere due to confinement is a common phenomenon according to participants. This tension causes discomfort to patients, who react aggressively to others, resulting in increased rates of conflict and violence.

“We do not want the hospital to look like a prison, but unfortunately this is how patients perceive it. Doors locked, railings and nurses-guards.” (L1)

“The tension of confinement is so high that it often results in aggressive behavior which needs to be restrained.” (L9)

Furthermore, several participants stressed that people have less trust in nurses in locked spaces and even when this happens it takes a long time to be established. Apart from the suspicion characteristic of several mental health service users, this situation is aggravated by the practice of door locking that makes “nurses” look “bad” in the eyes of service users.

"Imagine going to a house and suddenly the host locks the door and forces you to stay inside. That's exactly how patients see it. How easy is it to trust us afterwards? " (L13)

Some of the nurses pointed out that one of the disadvantages of locked spaces is patients’ resignation, passivity and dependency. They emphasize that when patients have care on a 24-h basis their recovery is hindered. Treatment not founded on cooperation prevents people from taking responsibility for themselves. In this way service users gradually get disempowered. Some of the nurses underlined the stigma towards people and nurses themselves.

“In the long run, the patients’ stay in the ward for a long time I think negatively affects them because they are comfortable, rested and stop taking initiatives.” (L11)

Most nurses agreed that symptoms of compassion fatigue are related to the atmosphere of the working environment and the distancing from people who face the dual burden of mental health problems and trauma. Many stressed the importance of the integrity of professional’s personality in order to cope with the difficult situation that he/she often has to face.

“You are very tired mentally. You do not want to talk to for two hours. Many times I come home exhausted, my head is buzzing, I need to calm down or a pill to sleep.” (L13)

“The psychiatric hospital can pressurize you psychologically, it can darken your soul as we say. Especially when you spend half your day looking at walls and locked doors. After the end of my shift I always try to forget everything and calm down. During the shift I often distance myself from patients to control my emotions and to protect myself psychologically.” (L15)

### Theme 4: the revolving door

The interviewees described the revolving door as a source of compassion fatigue for nurses and barrier to recovery for service users related to lack of care continuity after discharge and substance misuse during admission and after discharge. Sub-themes were (a) service users’ relapse and nurses’compassion fatigue, (b) lack of care continuity, (c) substance misuse, (d) limited resources and containment in the context of multidisciplinary team, and (e) guilt and fear of litigation.

Participants had the opportunity to describe their life inside and outside the hospital from the moment they first started working to the point of data collection. Frustration, tension and compassion fatigue were words that they used during the interviews. The biggest concern of several nurses was the re-admission of patients. Their comments showed their frustration and indignation when they had made a great effort to support a patient and he/she returned after a while. Some nurses stressed that lack of care continuity and collaboration with mental health professionals in the community hinders service users’ recovery.

“Sometimes I find myself trying hard for a patient. I feel so proud to see him/her leave and be happy and thank me for the care provided by the unit. I cannot explain the frustration I feel when I see him re-admitted. Most of the times my efforts are thwarted. A few times you get satisfaction. My biggest fear is to see patients who have made progress coming back. I get upset and I feel all our effort are cancelled, especially when the patient is young … I started with dreams and hopes to change a lot but in the process I compromised. With time I realized that no matter how much soul I put in, there are not appropriate structures in community and help from the state. “(O1)

“The community is indifferent to service users’ effort to work on their recovery after being discharged. It does not have the proper infrastructure to receive these people and help them stand on their own feet. When there is no one in the community to care for them, to help them get their medication or support them at some point they will go back to the hospital to get help.” (L12)

Furthermore, the import of psychoactive substance users constitutes a considerable burden on the nursing work and appeared to provoke intense negative reaction to participants.

“I get very angry when I have patients who use and are in the unit just because they are users. We do not help them as long as the department is open and they continue to use.” (O8)

“What makes me tired is the re-admission of users. And they come in again if the department is open; if he/she wants to find his/her dose, he/she will find it, no one is stopping him/her”. (O9)

Inadequate patient activity during hospitalization is reported as a major disadvantage in patient’s recovery. Nurses report that there are not enough activities for patients during their treatment in the unit. Low staff levels and inadequate staff education on working with groups contribute to low levels of patients’ activities. However, in the open wards, the patient has the opportunity to socialize with other patients during the day. Socialization works therapeutically and the contact with other patients covers to some extent the lack of activities and occupational therapy.

“The biggest disadvantage of the locked unit is that it is not fully staffed, it needs more staff, it needs occupational therapists … but also other professionals so that the patients are involved in some activities. They cannot lie down all day, nor watch TV all the time, it is not good for their mood but also for their health. This way we will be able to work with patients within a nursing approach and create a therapeutic relationship with them. If this happens there will be less tension … ” (L10)

Several participants mentioned that lack of resources included staffing levels, support from managers, clinical supervision and employment of security personnel so as to ensure that nurses are not burdened with the locking and unlocking demands on unit entrance. Furthermore, they went on to emphasize that they felt exposed by the legal framework because they are considered accountable and burdened with lengthy legal proceedings that affect them in both their professional and personal lives. Participants often felt guilty during their everyday work life.

“You have a big share of responsibility because you are also locked in here and usually everything is the fault of the nurse on shift.” (L9)

## Discussion

Overall, in the present study we identified four types of doors in nurses’ experiences of their working environments: the open doors, the invisible doors, the restraining doors and the revolving doors. Open doors symbolised trust, therapeutic opportunity, respect and shared decision making. Leaving the door open appeared to be a simple but symbolic anti-stigma act of social inclusion against ‘othering’ social processes [47] related to archaic fears towards mental illness [48]. Similarly, invisible doors symbolized permeability of spaces in psychiatric care [18, 49] and appeared to satisfy symmetrically safety and care imperatives by collective containment of stress among staff [2]. In contrast, the restraining doors symbolised loss of control, social distance and stigma which seems to be echoed in the recent impact of lock-downs during the Covid-19 pandemic demonstrating that restriction of the crucial control over space [50] is associated with considerable increases in aggressiveness, violence, substance misuse, trauma and social isolation within families, groups and communities [51]. Furthermore, the restraining doors appeared to deprive nurses and service users of the necessary feeling of safety to engage in therapeutic encounters imposing an atmosphere of fear [52] potentially leading to further restrain of the latters’ crucial control over space, recalcitrance or abscondence [53]. Finally, the revolving doors appeared to symbolise service users’ rejection of the offered therapeutic environment and mental health professionals’ failure in their professional role sometimes personified, projected or attributed to service users in the expression ‘the revolving door patients’. Furthermore, the revolving door was a source of deep feelings of frustration and indignation for nurses in both open and locked wards and was linked to similar attributions in both environments related to internal microsystem acute care aspects (limited staffing levels, support, recourses and activities for service users) as well as ‘locked doors’ in the community (limited care continuity, stigma, organisation of community mental health centres with limited number or no mental health nurses).

Trust given to people indirectly by the open doors contributed, according to participants, to therapeutic engagement and shared decision-making processes which in turn facilitated containment of mental health crisis and service users’ self-empowerment. In essence open doors were described as compatible with person-centered and recovery-oriented care while emphasis on ward atmosphere is similar to findings of previous research [8]. Furthermore, strenuous discussions were necessary to build communication bridges with people and their families as in the case study of Di Napoli and Andreatta (2014). Nonetheless, Di Napoli and Andreatta [54] described an acute care environment operating on a non-restraint protocol. In the present study, only one open unit operated on a non-restraint protocol [55]. All other open acute care spaces achieved containment of mental health crisis primarily through a multidisciplinary team approach and employed restrictive measures as a last resort.

As containment was embedded in the context of an acute ward, inevitably nurses had to be directive and coercive in some instances [56]. Björkdahl et al. [57] found containment by control, coercion or force to be a therapeutic act. This may be because it was conducted on a psychiatric intensive care unit, where the most violent and aggressive individuals are cared for, hence control, coercion and force were necessary to maintain the physical safety of some individuals. The rest of the literature spoke of containment by control as a last resort, and on the whole, it was considered non-therapeutic [7]. Technical safety features strongly in measures to reduce risks of absconding or self harm. Nursing practice is influenced by ‘expert’ views, imposed at central institutional level, on particular risks to be avoided. These included: escape from the forensic wards of mental health patients subject to Ministry of Justice restrictions, cases of suicide involving shower rails or curtains failing to meet prescribed standards, or deliberate or accidental falls from unrestricted windows [58]. An emphasis on technical procedures and rules to enhance security and safety for staff and service users and the general public, may make it difficult to provide recreational, psychotherapeutic, educational, spiritual and occupational therapies [59]. While a ‘safe place’ implies a reasonable degree of ‘technical safety’, it may, as importantly, embrace social, psychological, and emotional safety, corresponding to the relational, social, and symbolic dimensions of therapeutic landscapes [58].

As regards the theme “invisible doors”, it denotes locked doors in acute care spaces which depict a physical and a spiritual closeness between staff members and people that gives the latter peace [60]. It appears that an overall positive ward atmοsphere in a rich social environment, caring and respectful informal interactions and openness between mental health professionals and people can cultivate a sense of freedom in an acute psychiatric care unit and render it ‘permeable’ [18, 49].

On the other hand, the restraining door descriptions include nurses’ recognition of and concern for people’s negative feelings and reactions such as tension, aggression and physical injury which ultimately result in feeling uncontained and dissatisfied with care. Even when the door locking was considered as a necessary part of the work, discomfort with a time-consuming task blurring their professional role and hindering the building of therapeutic alliances was prominent and similar to that of other research [21]. Structural and cultural characteristics of the psychiatric hospitals of the present study [61], low staff levels and resources due to the Greek economic crisis of the last decade [42], previous traumatizing experiences of involuntary hospitalization [62, 63] may have also impacted the attempts of mental health nurses in the present study who strived to find space for therapeutic engagement.

Finally, according to participants the revolving doors which were mostly attributed to lack of care continuity after treatment, lack of activities and substance misuse during in-patient care appeared to be related to nurses’ feelings of frustration and compassion fatigue. Economou [41] in a study of mental health professionals to severe mental illness in the two Psychiatric Hospitals of Attica reported that unfavourable attitudes to severe mental illness were limited to pessimism about recovery, difficulty in viewing people with mental illness as similar to other people and desire to keep distance in intimate encounters. Economou et al. suggest that their findings, although aligned to international findings, may reflect staff burn-out or could be attributed to the chronic and usually revolving-door service users found in the psychiatric hospitals of Attica. Indeed, increased rates of secondary traumatic stress among psychiatric nurses and/or different mental health nurse profiles [14–16] may explain the present study participants’ distancing from people as a means to protect themselves [38].

Curtis et al. [58] emphasized how responsibility for technical safety was being invested in the physical infrastructure of certain ‘places’ within the hospital where risks are seen to be ‘located’. Staff seemed to feel that in relying on technical safety measures they were, to a degree, divesting themselves of human responsibility for risks they are required to manage. If carers are to be seen as equal partners in the treatment and recovery of mental health service users, then as well as being aesthetically pleasing, safe and secure, it is important that the hospital environment be experienced as ‘permeable’ for them in their caring role [64]. Carers and family members need to have access to a variety of different settings within the hospital where they can spend time with a patient during their visit; private living rooms and garden spaces similar to those enjoyed in the domestic family home [64]. A holistic understanding of the essential components of containment and therapeutic relationships in acute psychiatric care spaces and the symbolic dimensions of doors, may allow both staff and service users to reach their potential.

### Limitations

As regards the limitations of the present study, the sample was drawn at two psychiatric hospitals only, and therefore may not be representative of nurses in Greece in general. Furthermore, interviews with nurses with sustained exposure to psychiatric practice in other hospitals, would allow comparison of perceptions and experiences which would not be influenced by professional socialization processes at a particular hospital. Finally, although the sociodemographic and professional characteristics of nurses in this study were very similar to those of participants in other studies evaluating attitudes and characteristics of the nursing practice environment in other countries, the fact that other characteristics of wards’ culture were not included constitutes another limitation of the present research.

#### Implications

If nurses experience ethical dilemmas related to their practice then there is a clear need to cultivate and retain a critical and analytical attitude towards the system they operate [2]. Clinical supervision may support mental health nurses at an individual and team level in this challenging task. A multidisciplinary approach is of utmost importance in achieving continuity of care and containment of personal suffering. Addiction and trauma education might also enable mental health nurses to provide care to people who exhibit challenging behaviours. Research into people’s perceptions of treatment and personal recovery might inform service provision in open and locked wards. Finally, an urgent increase in mental health nurse staffing levels is required to avoid an increase in the use of locked doors and rigid demarcated spaces in acute care wards. In a context of increased demand for services, funding difficulties and staff shortages further complicated by the recent COVID pandemic and the severe impact of the global financial crisis, open systems and permeability of mental health care spaces is crucial to resist the decline of therapeutic mental health landscapes, contain mental health suffering and instil hope, connectivity, meaning and empowerment of persons with mental health problems.

## Data Availability

Τhe dataset supporting the conclusions of this article are available from the corresponding author upon reasonable request.
